# Comparison of two different methods of colon cleansing for afternoon-colonoscopy

**Published:** 2014

**Authors:** Kobra Baghbani, Javad Shokry-Shirvani, Hassan Taheri

**Affiliations:** 1 Department of Internal Medicine, Ayatollah Rouhani Hospital, Babol University of Medical Sciences, Babol, Iran.

**Keywords:** Colonoscopy, Colon, cleansing, PEG solution, PM, AM

## Abstract

***Background: ***The appropriate colon cleansing is a major determinant of quality of colonoscopy. This prospective randomized study was designed to compare the efficacy and tolerability of the morning (AM) PEG (polyethylene glycol) solution to previous-evening (PM) PEG solution for the afternoon colonoscopy.

***Methods:*** This comparative study compared the AM to PM prep for afternoon outpatient colonoscopy. The subjects randomly received PEG PM dose ;4 liters of water plus 4 pack PEG powder at 6 pm before colonoscopy (250 ml every 15 min) or AM ( the same dose solution at 6 am on the day of colonoscopy). The preparation and colonoscopy quality, PEG side effects, sleep quality, lesion detection, flush need and suction fluid were compared in these two groups.

***Results:*** One hundred seven cases received AM prep and 102 received PM prep. The colon prep was adequate in 94.4% in AM group and in 90.2% cases in PM group (P=0.2). The incidence of adverse events in these two groups was similar. Sleep quality and the need for flush was lower in the AM group (P=0.004 and P=0.03). The mean volume of suction fluid was higher in the AM group (P=0.01). The detected lesions were similar between the two groups. Adequate prep was associated with lower flush need in AM group (P=0.001).

***Conclusion: ***AM and PM PEG solutions were clinically equivalent with cleansing efficacy and side effect and lesion detection. AM group was associated with a better sleep quality and less flush need, but more suction fluid.

Colonoscopy is the preferred procedure for the investigation of large bowel and terminal ileal disease in adults and children (1-2). Effective bowel preparation is important for adequate examination of the colon during colonoscopy (3-6). Inadequate cleansing can result in missed pathological lesions (7). There are different protocols for bowel cleansing. Two of these schedules are used, PM dosing in which the PEG is administered the night before colonoscopy and AM dosing in which the prep is given on the day of colonoscopy (4, 7, 8). There may be some advantages in using the AM dosing schedule for colonoscopy, the prep and procedure, is a 1-day process. The AM schedule has the potential to minimize the prep’s interference on the patient’s ability to work and sleep the day before (9, 10). In addition, the patients perceive AM dosing to be better tolerated as the predicted adverse events occur the day of procedure as compared with PM dosing over 2 days (9-14). Since 1980, polyethylene glycol (PEG) solutions have become the most commonly used laxatives for colon cleansing (12-13). Using PEG solution does not require supplemental laxative (11-15). 

The purpose of this study was to compare AM PEG with PM dosing for achieving adequate cleansing for performing colonoscopy.

## Methods

From March 2013 through August 2013, patients aged 18-80 years scheduled to undergo elective outpatient afternoon (1pm or later) colonoscopy at two teaching university hospitals were entered in this study. The aim of this study was to compare the efficacy and tolerability of morning-only (AM/PEG) solution to previous-evening (PM/PEG) solution for afternoon colonoscopy. A written informed consent was obtained. From each patient Exclusion criteria included pregnancy, breast feeding, >50% colon resection, severe constipation (<1 bowel movement at week), known or suspected gastroparesis, severe nausea or vomiting, bowel obstruction, heart and renal failure.

Using randomization, the eligible patients were assigned to AM and PM PEG groups by an investigator not involved in the colonoscopy procedure. The subjects were provided with a standard PEG solution and routine preparation for colonoscopy including soft liquid. The patients’ endoscopist was not involved in the randomization process and remained blinded to the patients’ preparation schedule in the duration of study, all study procedures were performed by an attending physician. The subjects randomized to PM dose PEG were instructed to take 4 liters of water plus 4 pack PEG powder at 6 pm, the afternoon before colonoscopy (250 ml every 15 min). The subjects assigned to AM group took 4 liters of water plus 4 pack PEG powder at 6 am on the day of colonoscopy. All patients received 20 mg bisacodyl the day before colonoscopy. Diet instruction was identical for both study groups, one day before colonoscopy, the patients were allowed to a low –residue breakfast and then soft liquids diet. On the day of colonoscopy, the patients completed a questionnaire evaluating sleep, life and work quality and the side effects of PEG. Life and work quality was measured using a 10-point Likert scale and the patient’s side effects (nausea, vomiting, abdominal pain, light headedness and anal pain) were registered. Sleep quality was measured by the average number of hours that the patient normally slept the night before colonoscopy. During the procedure, the endoscopist recorded the total procedure and time of procedure, lesions and bowel site intubation. During the procedure, the endoscopist graded the prep quality as follow: Excellent= small amounts of clear liquid; Good= Residual liquid stool, all mucosa seen; Adequate= some particulate matter, >90% of mucosa seen; Poor= Substantial particulate matter of solid stool, <90% of mucosa seen. The estimated amount of flush was defined as: none, <50, 50-100, >100 cc). The volume of the suction fluid was measured as well. The primary trial outcome was the measurement of the quality of preparation (adequate vs inadequate) for the colon. Adequate was defined excellent good and or fair preparation, and inadequate poor preparation. The flush need and suction of fluid, prep side effects, duration of procedure, sleep quality and colonoscopy finding were the secondary outcomes. 

**Statistical analysis: **The data were collected and analyzed using SPSS Version 18. The quantitative variables were compared by t-test and the qualitative variables by chi-square test in these two groups. The study was designed to have 80% power (with α=0.05 and target sample size 172). A p-value <0.05 was considered significant.

## Results

During the study period, 209 outpatients were randomly selected to AM or PM prep. One hundred seven patients (63 females, 44 males) received AM prep and 102 (56 females, 46 males) received PM prep. The mean age of PM group was 48.32±14.23 and the AM group was 48.52±14.48 years (P=0.91).The colon prep was adequate in 101 (94.4%) patients in the AM group and in 92(90.2%) patients in the PM group (P=0.2).The cecum and terminal ileum was intubated in 94 (87.8%) patients in the AM group vs 84 (82.8%) patients in the PM group that was not significantly different between groups (P=0.33).

The withdrawal time, which consisted of the time spent inspecting the mucosa ranged from 6 to 38 min ,with median of 11.6+3.9 min in the AM group vs 11.8+4.9 min in the PM group (P=0.65). In the AM group, 96(89.7%) of patients were required < 100 cc fluid for flush vs. 80 (78%) in the PM group. The flush need in AM group was significantly less than PM group (P=0.036). The suction fluid during the procedure was 191+ 100 cc of AM group vs 142 +107 cc for PM group. The suction fluid was lower in PM group versus AM group (P=0.01). Adequate prep was associated with lower flush need (P=0.001). There was no correlation between suction fluid and prep quality (P=0.08). The overall incidence of side effects was not significantly different between the two groups (P=0.63) (table 1). Duration of sleep the night before colonoscopy in the AM group was 6.2+1.2 hours vs 5.5 +1.5 hours in the PM group and AM group had better sleep quality than the PM group (P=0.004). The quality of life in the AM group was 9.6 +0.74 from 10 score vs 9.5+0.74 in the PM group (P=0.17). The colonoscopic findings were similar in AM and PM groups (P=0.65) (table2).

**Table 1:** PEG complications in AM and PM preparation groups

**Complication**	**Group(n=209)**		**p-value**
	**AM(n=107)** **N(%)**	**PM(n=102)** **N (%)**	
None 135 (64.6%)	75 (70)	60 (58.8)	0.08
Nausea 41(19.6%)	19 (17.8)	22 (21.6)	0.46
Abdominal pain 21(10%)	8 (7.5)	13 (12.7)	0.2
Lightheadedness 7(3.3%)	3 (2.8)	4 (3.9)	0.8
Vomiting 3(1.4%)	1 (0.9)	2 (2)	0.9
Anal pain 2(1%)	1 (0.9)	1 (1)	0.9

PEG: polyethylene glycol, AM: Ante Mortem, PM: Post Mortem

**Table 2:** Colonoscopy findings in these two groups of the study

**Lesion**	**Group (n=209)**		**P-value**
	**AM (n=102)**	**PM (n=107)**	
Normal 100(47.8%)	54 (50.5)	46 (45.1)	0.43
Polyp 30(14.4%)	15 (14)	15 (14.7)	0.9
IBD 21(10%)	12 (11.2)	9 (8.8)	0.56
Diverticulum 9(4.3%)	2 (1.9)	7 (6.9)	0.07
Mass 10(4.8%)	4 (3.7)	6 (5.9)	0.46
SRU 2(1%)	1 (0.9)	1 (1)	0.9
Other 37(17.7)	19 (17.8)	18 (17.6)	0.9

PEG: polyethylene glycol, AM: Ante Mortem, PM: Post Mortem

## Discussion

The diagnostic accuracy of colonoscopy depends on the quality of bowel preparation and inadequate mucosal visualization may lead to miss premalignant lesions. Patient compliance and tolerability of the preparation method is also a very important factor because the patient’s ability to complete the cleansing program impacts on a successful colonoscopy. The third factor influencing any achievement to desired colonoscopy is safe cleansing programs.

In this study, we aimed to find oral PEG administration the best time schedule. We showed that AM group had 94.4% adequate prep vs 90.2% in PM group. We believe that the AM administration of PEG provides a good quality of cleansing and diagnostic yield and prevents repeat examination.

Overall in this study, the incidence of the side effects were not significantly different between the two groups and it could be said that the method of bowel prep a few hours before the examination would have impact on the patient's activities during a shorter amount of time. The lesion detection, procedure time and quality of life was similar in the two groups. In this study, the duration of sleep was better in the AM group than the PM group and flush need was lower in the AM group. Adequate prep was associated with lower flush need. Although suction fluid was higher in AM group but did not interfere with the adequacy of colon cleansing. 

In 2010, Matro conducted a study on 125 patients, the colon prep was adequate in 92% AM preps vs. 94% the AM-PM group. The polyp detection was greater in the AM group, the incidence of adverse events was not significantly different between the two groups, but the AM group had lower incidence of abdominal pain, the AM group also had better sleep quality and less interference with the previous work day (9). 

These findings were obtained in Gupta’s study in 2011 and Varghes’s study in 2010 (16, 17). In another study in 2006, Parra Blanco compared two different preparation methods on a same day protocol on 177 patients, the subjects received PEG or sodium phosphate on the same day or the day before schedule. The patients on the same-day group obtained good to excellent global cleansing scores more frequently than the patients who received PEG or sodium phosphate on the day prior-to-the procedure. Flat lesions were more frequent in patients prepared on the same day (4). 

Our study showed that AM and PM administration strategies for PEG are clinically equivalent to prep quality, procedure time and lesion detection. Furthermore, AM dosing was superior to PM dosing with less sleep interference before colonoscopy and flush need. An important limitation of our study was that some information obtained used self-report for example on the side effect considerations because the answers were subjected to sedative drug influence which interfere with the recall of some side effects. The second limitation is that the AM group had longer duration of colonoscopy due to time spent for the suction of the remaining fluid in the colon. Third, we did not exclude or adjust the patients with severe constipation and this unmeasured factor might affect the result. According to this study, PEG can be administered in the morning on the day of colonoscopy. AM dosing is a viable option that moves the process of colonoscopy (prep and procedure) towards becoming a one –day process.

## Notes

**Funding:** The Research Council of Babol University of Medical Sciences financially supported this study (Grant Number: 9135212).
